# Identifying barriers and facilitators along the hepatitis C care cascade to inform human-centered design of contextualized treatment protocols for vulnerable populations in Austin, Texas: a qualitative study

**DOI:** 10.1186/s43058-023-00484-6

**Published:** 2023-08-17

**Authors:** Anmol Desai, Lauren O’Neal, Kia Reinis, Cristal Brown, Michael Stefanowicz, Audrey Kuang, Deepak Agrawal, Darlene Bhavnani, Tim Mercer

**Affiliations:** 1 Department of Population Health, The University of Texas at Austin Dell Medical School, Austin, USA; 2 The University of Texas at Austin Dell Medical School, Austin, USA; 3 Department of Internal Medicine, The University of Texas at Austin Dell Medical School, Austin, USA; 4 CommUnityCare Health Centers, Austin, USA

**Keywords:** Hepatitis C, Homelessness, Intravenous drug use, Qualitative research, Human-centered design, Implementation research

## Abstract

**Background:**

Hepatitis C virus (HCV) is a leading cause of liver-related mortality and morbidity. Despite effective direct acting antivirals and a simplified treatment algorithm, limited access to HCV treatment in vulnerable populations, including people experiencing homelessness (PEH) and people who inject drugs (PWID), hinders global elimination. Adapting the evidence-based, simplified HCV treatment algorithm to the organizational and contextual realities of non-traditional clinic settings serving vulnerable populations can help overcome specific barriers to HCV care. The first phase of the Erase Hep C study aimed to identify barriers and facilitators specific to these vulnerable populations to design the site-specific, simplified treatment protocols.

**Methods:**

Forty-two semi-structured qualitative interviews, guided by the Practical, Robust Implementation and Sustainability Model (PRISM) framework, were conducted with clinic staff, community-based organizations providing screening and linkage to care, and patients diagnosed with HCV, to identify contextual barriers and facilitators to treatment at a local community health center’s Health Care for the Homeless program in Austin, Texas. Audio-recorded interviews were systematically analyzed using thematic analysis informed by the PRISM framework and design thinking, to anchor barriers and facilitators along the HCV care cascade. Findings were fed into human-centered design workshops to co-design, with clinic staff, site-specific, simplified HCV treatment protocols.

**Results:**

The specific needs of PEH and PWID patient populations informed barriers and facilitators of HCV care. Barriers included tracking patients who miss critical appointments or labs, medication access and adherence, and patient HCV knowledge. Clinical teams leveraged existing facilitators and incorporated solutions to barriers into clinic workflows to improve care coordination and medication access. Actionable solutions included augmenting existing staff roles, employing HCV care navigation throughout the cascade, and standardizing medication adherence counseling.

**Conclusions:**

Clinic staff identified HCV care facilitators to leverage, and designed actionable solutions to address barriers, to incorporate into site-specific treatment protocols to improve patient HCV outcomes. Methods used to incorporate staff and patient experiential knowledge into the design of contextualized treatment protocols in non-traditional clinic settings could serve as a model for future implementation research. The next phase of the study is protocol implementation and patient enrollment into a single-arm trial to achieve HCV cure.

**Supplementary Information:**

The online version contains supplementary material available at 10.1186/s43058-023-00484-6.

Contributions to the literature

• Global elimination of hepatitis C virus (HCV) requires expanding treatment and targeting vulnerable populations, including people experiencing homelessness (PEH) and people who inject drugs (PWID).
• Clinics serving PEH and PWID require simplified, site-specific HCV treatment protocols to improve patient HCV outcomes.
• Obtaining a broad, multi-level perspective of barriers and facilitators to HCV treatment using qualitative methods grounded in an implementation science framework allows for design of site-specific HCV treatment protocols.
• We offer a model for future implementation researchers to design contextualized care protocols for treating vulnerable populations in non-traditional clinic settings.
• We also offer a methodology for change management for clinic leadership and staff to use human centered design and experiential knowledge from staff to improve workflows or when rolling out new protocols.

## Background

Chronic hepatitis C virus (HCV) infection affects 58 million people globally and 2.1 million people in the United States (US) [1]. HCV incidence in the US is rising and remains a significant driver of liver-related morbidity and mortality [2]. Ongoing HCV transmission among people who inject drugs (PWID) and increased risk of acquisition and transmission among people experiencing homelessness (PEH) in the US has the potential to sustain the epidemic [3, 4]. Among PEH, estimated HCV prevalence is more than 30%, though estimates are often underreported [5]. The prevalence of HCV is 31% among those seeking care at Health Care for the Homeless (HCH) clinics, and 70% among those seeking care at HCH clinics who inject drugs [6].

Despite direct acting antivirals (DAAs) and a simplified HCV treatment algorithm with a > 97% cure rate, only a minority of the total population living with chronic HCV, who are aware of their status, has access to care (43%) or has been prescribed HCV treatment (16%) [7, 8]. In particular, access to treatment among vulnerable populations remains low [1, 9, 10]. PEH can face challenges to HCV care due to the stigmatization of the population and their transient nature, making it hard to keep appointments or maneuver long appointment wait times [9]. Access to treatment is obstructed by other patient-level and systemic barriers, including misconceptions about HCV treatment, a limited number of experienced HCV providers in primary care clinics, payor restrictions, and overly complex organizational workflows [9, 11, 12].

To increase access to treatment among PEH and PWID, clinics that serve these vulnerable populations urgently need locally adapted and contextualized protocols that use DAAs and the simplified treatment algorithm, while addressing patient and systemic barriers to, and facilitators of, HCV care. In order to contextualize HCV treatment protocols that address multi-level barriers to HCV care in Austin, Texas, we explore a combined perspective of patients, clinic and system level staff, and external HCV testing staff, unlike other studies that focus mainly on one perspective. Additionally, we go one step further than other studies that tend to focus on presenting the barriers and facilitators only, by aligning the barriers and facilitators along specific steps of the HCV care cascade and presenting operational solutions to address these barriers through human-centered design.

The purpose of the Erase Hep C study is to develop and evaluate the implementation of site-specific HCV treatment protocols, which is detailed in our published protocol paper [13]. We split the study into two phases, where in the first phase we utilized qualitative research methods guided by an implementation science framework combined with human-centered design thinking, to develop the site-specific HCV treatment protocols by identifying and reducing barriers and leveraging facilitators of HCV care.

## Methods

### Study design

**Fig. 1:**
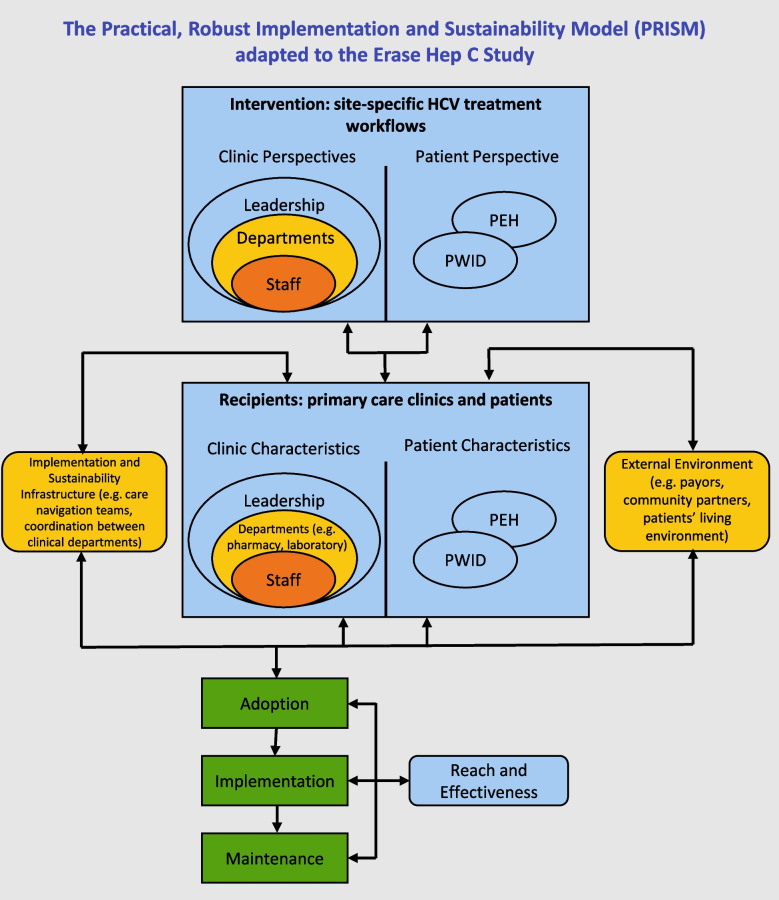
PRISM implementation science framework adapted to the Erase Hep C study

We used a human-centered design approach, going through two of the four cyclic phases in this first phase of our study: discovery and design [14]. The Practical, Robust Implementation and Sustainability Model (PRISM) framework was used in the discovery phase to inform development of semi-structured qualitative interview guides to identify multi-level contextual barriers and facilitators to HCV treatment in our community health center (CHC) system’s high-risk, vulnerable patient population (Fig. 1) [15]. Though a multitude of implementation science frameworks exist, the PRISM framework was best fit for this study as an extension of the RE-AIM framework, one of the most used, which we plan to use in the second phase of our study [16, 17].

We leveraged knowledge and experience from patients diagnosed with HCV, clinic staff and providers, our CHC system’s leadership, and community-based organizations providing HCV testing and linkage to care, to inform design and implementation of simplified, site-specific HCV treatment protocols. We chose interviews over focus groups to elicit individual level perspectives about organizational structures and identify tasks that individuals perform. Interviews allowed people to speak freely and independently, which may have been affected by power dynamics in a focus group setting.

### Setting

Qualitative interviews were conducted in seven primary care clinics within our CHC’s HCH program and among external organizations that engage in the community to provide HCV testing and linkage to care. The clinics included the following: (1) a full-spectrum clinic located within the Austin Resource Center for the Homeless (ARCH) shelter (“the ARCH Clinic”); (2) a patient-centered brick-and-mortar clinic providing full-spectrum care for PEH as well as other medically complex and socially vulnerable patients following hospital discharge (“Care Connections”); (3) a clinic dedicated to providing medication-assisted therapy (MAT) to individuals with opioid use disorder (“the MAT Clinic”); (4) a clinic located at Community First! Village (CFV), a permanent supportive housing community for individuals who were formerly chronically homeless (“the CFV Clinic”); (5) a clinic located at a state-sanctioned encampment for PEH that is becoming a transformational shelter complex (“the Esperanza Clinic”); (6) a full-spectrum clinic space set-up within Sunrise Church, providing care alongside other partners of the Sunrise Homeless Navigation Center non-profit that serves PEH in Austin (“Sunrise”); and (7) a street medicine team bringing care to PEH at homeless campsites and under bridges (“the Street Team”). The Mobile Team operates three of these HCH clinic sites: CFV, Esperanza, and Sunrise. These HCH clinics serve people currently or previously having experienced homelessness, or people who inject drugs (PWID), providing both primary care and connection to a variety of social resources.

### Participants

We interviewed a total of 42 people, made up of 28 clinic staff and providers, 10 patients diagnosed with HCV, and 4 staff from external organizations that screen or link patients with HCV to care. To engage clinic staff across all levels of our CHC, we used a purposive sample to ensure we had a wide representation of roles that participate in HCV care and interact with our population within each clinic, with a similarity of roles across clinics [18]. We applied the same sampling method for external agencies. We used a convenience sample to engage patients, by asking providers and staff to recommend patients with HCV, from treatment naïve to having achieved cure, who would be willing to participate in our interviews [18].

### Data collection

A trained research coordinator and research assistant conducted thirty to sixty-minute audio-recorded interviews in English. Interviews with staff were conducted both in person and over Zoom. All interviews with patients were conducted in person at the clinic to receive a warm hand-off from the clinic team to help build trust between the interviewer and interviewees. The research coordinator and research assistant were not known to the patients and were only known to a few clinic staff, to the extent that they were informed of the study and were given a guided tour of the clinical sites. For the patient interviews, warm hand-offs from clinical staff to the research staff were used to make the patient feel more comfortable. Interviews were conducted between September and November 2021. Participants were asked about the process to initiate HCV treatment, challenges to starting and completing treatment, and facilitators of treatment adherence and completion. Staff, providers, and leadership were also asked about their role in the process of HCV care for patients. If additional and relevant information surfaced, conversations progressed beyond the semi-structured interview guides. At the end of each interview, the participants were asked demographic questions. We conducted interviews until we reached thematic saturation of data and perspectives. Data collected from the interviews were supplemented with observations and discussions of clinic processes with clinic teams at each clinic site that were conducted in July 2021.

Written consent was obtained from participants, either in person or digitally. Participants were compensated $10 worth for being interviewed. Signed consent forms, audio-recordings, and transcribed interviews were all stored on Box, a HIPAA-compliant cloud storage system. This study was approved by the Institutional Review Board at the University of Texas at Austin and the Research and Quality Improvement Committee at CommUnityCare community health centers.

The research team consisted of a research coordinator with extensive experience in global health fieldwork conducting large-scale nationally representative population and public health surveys; a research assistant with experience in HCV, qualitative research, and implementation science research; co-primary investigators with extensive qualitative, implementation science research, and field epidemiology experience; and a co-investigator who is a hepatologist, HCV expert, and health services researcher. The research team also included several CHC providers to provide context on treating vulnerable populations within these CHC clinic sites. This research was reported based on the Standards for Reporting Qualitative Research (SRQR) guidelines [19] (Additional File 1).

### Data analysis

All 42 interviews were transcribed verbatim, using a combination of Zoom transcription services and manual transcription, and anonymized for thematic analysis using NVivo 1.5.1 (QSR International, Burlington, Massachusetts) [20]. First, qualitative thematic descriptive and interpretive coding, informed by our PRISM framework, was used to ensure each level of the framework was addressed in analysis. Second, a human-centered design thinking approach was utilized, focusing on the utility of the multi-level perspective data to garner idea generation during the human-centered design workshops, to design site-specific, simplified HCV treatment protocols at primary care clinics within our CHC’s HCH program [14].

Two coders iteratively collaborated to define codes, finalize the codebook, highlight themes and subthemes of the barriers and facilitators, and identify relationships between themes. Themes focused on HCV treatment access at both the patient and clinic level, clinic processes both external and internal to the CHC system (e.g., across departments and other healthcare systems), and characteristics of the external environment that impact HCV patient outcomes. The codebook development was a hierarchical process with constant comparison, refinement of codes, and merging of existing codes [21, 22]. Coders initially conducted deductive code development, starting with a list of initial codes we expected to see emerge, based on our observations and guided tours of the clinical sites [20]. Coders entered the first round of coding with this initial codebook, in which they individually read and coded the same three transcripts. The coders came together to discuss, negotiate, and revise the codes. The coders then went on to code an additional five different transcripts and conducted a second round of discussion and revision. Transcripts coded in these first two rounds of coding were randomly selected to capture a wide scope of roles to develop the most representative codebook. The final code structure was developed by consensus after coding the remaining transcripts and by applying the perspectives and characteristics of the PRISM framework throughout [21].

### Design workshops

In the design phase of the human-centered design approach, findings from qualitative interviews were fed into iterative, site-specific design workshops held with clinic staff to ideate and adapt the simplified treatment algorithm into actionable, site-specific HCV protocols integrated within existing clinic workflows [14]. A total of nine site-specific workshops were held over two in-person iterations, with the Mobile Team discussing the three mobile sites as a collective (CFV, Esperanza, and Sunrise). A member of the patient Sexual Health Navigation team and the patient assistance program (PAP) teams participated in each workshop to contribute their insights on barriers and facilitators surrounding patient tracking and patient medication access, in addition to providing solutions that could be incorporated into site-specific protocols.

**Fig. 2:**
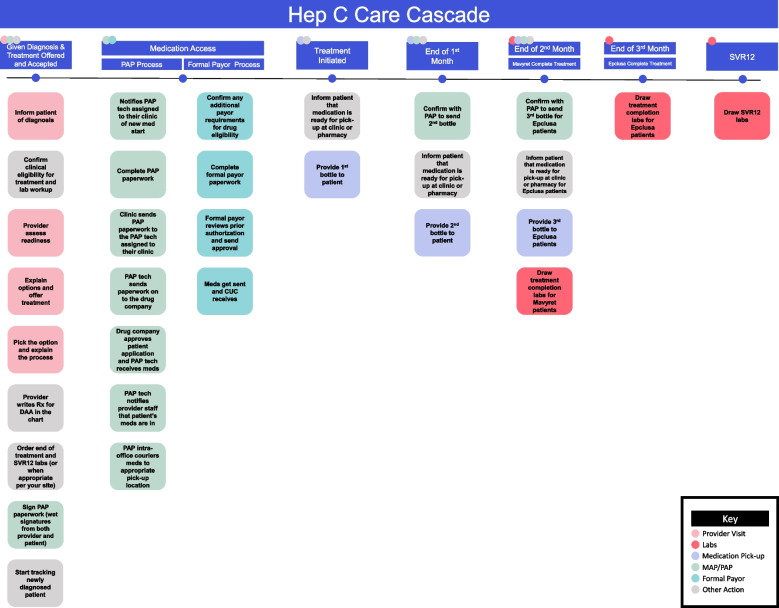
Hep C care cascade framework. Colored squares represent actions that occur along each step of the care cascade: provider visits (light pink), labs (hot pink), medication pick-up (lavender), medical access program (MAP) and patient assistance program (PAP) processes (green), formal payor processes (turquoise), otherwise synonymous with traditional commercial payors, and other HCV actions (gray)

Introductions were done at the start of the first workshops, followed by the workshop agenda. The first iteration of workshops discussed findings from the qualitative interviews with respondent validation and prioritization of barriers to address and facilitators to leverage with the site-specific protocols. After prioritizing barriers and facilitators specific to their sites, clinical teams focused on the design step of the human-centered design approach, ideating and conceiving actionable solutions to integrate into their site-specific protocol [14]. In preparation for the second iteration of workshops, providers and clinical leads simplified the HCV care cascade workflow (Fig. 2). Driven by the evidence-based simplified treatment algorithm, tasks no longer necessary were collapsed and removed, to define the HCV care cascade framework from diagnosis to cure.

In the second workshop, this care cascade framework was presented to and adapted for each site, considering their patient populations, non-traditional clinical environments, staffing ratios, and clinical workflows. The second workshops started with the agenda for the workshop and introducing and explaining the care cascade framework as the bare minimum of tasks required in the HCV treatment process. Clinic teams were given some flexibility to move tasks to different steps along the care cascade, if that was more efficient or effective for their site. Subsequently, the actionable solutions to mitigate the biggest barriers that were identified in the first workshop were layered onto the workflow. Clinic teams increased staff accountability by assigning ownership of each task to a role, with task sharing or role duplication to fit the flexibility and dynamic environment of each clinic. The actionable solutions addressed barriers and leveraged facilitators found across multiple PRISM perspectives and characteristics.

## Results

### Participant demographics

Forty-two participants were interviewed. Twenty-eight were clinic staff and leadership, including front-line clinical staff from each clinical site, as well as representatives from the referrals department, pharmacy department, and social and financial services departments (Table 1). We also interviewed four providers and system-level staff from other organizations in Austin, Texas, who screen and link people diagnosed with HCV and PEH to care. Ten patients were interviewed who were at different points along the HCV care cascade. Patients ranged from being treatment naïve to having been cured. Of the 10 interviewed, 4 were currently experiencing homelessness and 8 reported having injected drugs. All participants were native English speakers.

**Table 1:** Distribution of clinic and system-level staff interviewed

Classification of clinic and system-level staff interviewed	Number of staff interviewed
Providers (physicians (MD/DO), nurse practitioner (NP))	7
Clinic-level staff (registered nurse (RN), medical assistant (MA), medical administrative clerk (MAC))	8
Clinic-level social services staff (licensed clinical social worker (LCSW), community health worker (CHW))	5
System-level staff (pharmacy, referrals, social/financial services)	5
System-level leadership (manager, supervisor)	3

### Anchoring barriers and facilitators along the hepatitis C care cascade

**Fig. 3:**
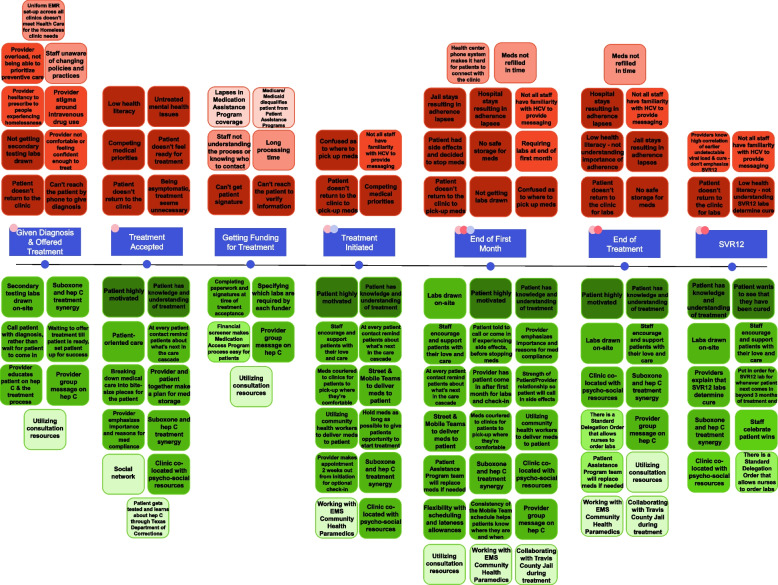
Barriers and facilitators along the hepatitis C care cascade. The dots represent patient actions along the care cascade (blue), as also indicated in Fig. 2, for which patients need to come for clinic visits (light pink), get labs (hot pink), and pick-up medication (lavender). The red squares indicate barriers with the shading gradient from darkest to lightest respectively representing patient-level, clinic-level, system-level, and environmental barriers. The green squares indicate facilitators with the shading gradient from darkest to lightest respectively representing patient-level, clinic-level, system-level, and environmental facilitators

By identifying barriers and facilitators to HCV care, clinic teams were able to conceptualize actionable solutions to barriers and leverage existing facilitators when designing the site-specific HCV treatment protocols. Barriers and facilitators were identified considering patient, clinic, provider, system, and external environmental characteristics and perspectives. The barriers and facilitators were anchored to every step along the HCV care cascade they impact, from diagnosis to confirmation of cure by sustained virologic response 12 weeks post-treatment (SVR12) (Fig. 3). Some barriers and facilitators affected multiple steps along the HCV care cascade and others were unique to specific steps.

The majority of barriers were patient-level while the facilitators were mostly clinic-level; however, many of the patient-level barriers may have been impacted by clinic-level barriers. Common themes at the patient level centered around the nature of the PEH and PWID populations, including transience, being vulnerable to theft, low health literacy, and difficulty adhering to medication and returning to the clinic for follow-up testing. Clinic and system level themes encompassed operational processes, including tracking patients who miss critical appointments, transporting medication to patients, enabling medication adherence, patient HCV knowledge, and failure to follow-up with patients for SVR12 laboratory tests confirming cure. Partnerships with external organizations facilitated the HCV treatment process by helping to find patients, aiding in building trust with the care team, taking medication out to patients, or drawing blood for laboratory tests. Table 2 provides illustrative excerpts of these themes, gathered from our interviews.

**Table 2:** Themes of barriers and facilitators identified along the hepatitis C care cascade, with illustrative excerpts from staff and patient interviews

Step in Hep C care cascade	Illustrative excerpt
**Given diagnosis and offered treatment**	
*Competing priorities*^A^	*“Sometimes with our patients, what you can do today is what you’re going to get done” (Clinic Staff)*
	*“Once in a great while I have a patient who says ‘I have a lot going on right now, I don’t really think I’m ready for it’.” (Provider)*
*Patient-Provider Relationship (waiting till patient can succeed)*^B^	*“I always offer it, but if they’re not ready, I don’t push. Because a lot of it is engagement, and I don’t want to scare them. So, if they’re on the fence, I can usually say ‘yeah, this is really good, knowledge is power, you should know what’s going on’ so you see which direction they’re in. But if they’re not ready, that’s totally fine. (Provider)*
**Treatment accepted**	
*Low health literacy*^A^	*“I didn’t know what it was…I didn’t know if it was serious or not. I had no information as to what it was, or what it can do to you. I was in the dark about it, so I didn’t care either which way because I didn’t know what it would do to me. I had no knowledge of what it is, so, it didn’t bother me much, but I was worried about it.” (Patient)*
*Patient motivation*^B^	*“I like my life. I want to get treated as fast as possible. I don’t [want to] infect anybody else…I want to get it over with.” (Patient who had been a PWID)*
*Breaking down the care cascade*^B^	*“I’ll go and I’ll speak to them, and give them this packet, go over, we have a timeline, a breakdown of like, you sign papers this day, you get labs this day, your medication this day. Go over with them what the program looks like.” (Clinic Staff)*
**Getting funding for treatment**	
*Cumbersome funding process*^A^	*“I think one of the major delays still is the time required for the prescription assistance program to kick in.” (Clinic Staff)*
*Medication coverage*^B^	*“Once they get [MAP], their visits are covered and medication is covered through the pharmacy. So that definitely keeps them going…I have this coverage that’s helping me get my medication, get my visits, so I’m here.” (Clinic Staff)*
**Treatment initiated**	
*Characteristics of the PEH and PWID populations*^A^	*“There’s the obvious barriers with patients experiencing homelessness, a lot of times they have coexisting mental health issues, substance abuse issues…mak[ing] it harder to remember that you have an appointment or remember to take your meds or having somewhere to secure your medication. Their stuff gets stolen, they lose things, they don’t have transportation, they don’t have phones. All those things with the homeless population are a huge barrier.” (Clinic Staff)*
*Medication replacement*^B^	*“[The patient] lost some of his [medication]. But he showed up right away, and we were able to get the drug company to replace them quickly enough to where I don’t think he missed very many days.” (Clinic Staff)*
*Corollary care*^B^	*“If you’re on suboxone [buprenorphine]…you want to live longer…you want to stop hurting yourself. So, I think…a lot of people will say, I want to get clean and sober because I don’t want to die or I want my body to be okay, I want my body to last. And so, I think it goes along with hep[atitis] C too. Like I want to take care of myself now.” (Patient)*
*Care environment*^B^	*“Everybody here, we have the same goals in mind for our patients, and really [want to] see them all succeed and get their treatment.” (Clinic Staff)*
**End of first month**	
*Medication adherence*^A^	*“…Reasons patients don’t complete treatment and some do experience side effects and don’t have anyone to talk to, or aren’t willing to complete it and stop…” (Provider)*
	*“The reason that people miss doses, or just stop entirely, will be because of some sort of side effect. The majority of the time, they’re not common side effects and I don’t really know if they even are true side effects of the medication, or just kind of something that happened around the same time, and the patient is just saying that that’s what it’s from.” (Clinic Staff)*
*Care coordination*^B^	*“If we saw them…remember you got to come in this day for your appointment and for your hep[atitis] C meds, your second bottle’s due…if I ever saw them walk by I would always remind them.” (Clinic Staff)*
*Patient-provider relationship*^B^	*“Please call me, so that you don’t have to suffer alone.” (Clinic Staff)*
**End of treatment**	
*Psychosocial resources*^B^	*“At CareConnections clinic there’s more resources: a counselor, wound care nurse, a foot doctor…a social worker, and a community health worker.” (Provider)*
	*“For the access of everything that’s being offered on a day-to-day basis, food, social service, and any other specified services that one may need, on his medical situation, mental health.” (Patient at Sunrise)*
**SVR12**	
*Returning to the clinic for labs*^A^	*“The single biggest reason for lack of SVR12 is the fact that patients don’t show up for the appointment. And I think that we as providers don’t do enough messaging on it, either. Most of us know that treatment completion labs, the last day of the treatment, portends treatment success, they’re highly correlated with SVR12.” (Provider)*
*Patient motivation*^B^	*“He tells me that I was cured. That I didn’t have [hepatitis C] anymore…That made me feel good! I achieved something…That’s the way I looked at it.” (Patient who achieved SVR12)*

A superscript^A^ indicates a barrier and a superscript^B^ indicates a facilitator

### Given diagnosis and offered treatment

#### Barriers

Several barriers centered around providers’ own comfort level in treating HCV, or in assessing treating PEH and PWID, assuming either they would not be able to adhere to treatment or would get re-infected, assumptions stemming from the stigma surrounding PEH and PWID. Additionally, some providers and clinic staff mentioned not always being aware of changing policies and procedures, which is a barrier to offering and initiating the hepatitis C treatment process.

“I wasn’t aware that laws have changed and there [were] different requirements…Because I just don’t know that so, I ordered the labs that I had previously ordered at [the Community Health Center] and this patient’s claim got rejected, and they [said] no, we need these other particular tests, and I [said] I didn’t know that. So, then, I had to have [the patient] come back and get labs drawn again and so that’s frustrating for the patients.” (Provider from External Agency)

Among providers who are comfortable treating HCV in this population, treatment may not be offered if they are experiencing provider overload, may not have sufficient time for educating patients, or may need to prioritize other acute medical needs.

“In the patient population that I was serving, some of that preventative care, when you triage how important it was, it wasn’t as important as keeping them alive and out of the hospital…if you’re constantly putting out a fire it’s hard to get to the preventative side of the care.” (Provider)

Lack of access to easy, comfortable, and reliable transportation and inability to contact patients due to low phone ownership were the most commonly reported obstacles to care, beginning with receiving an HCV diagnosis, and at every stage of clinic-patient interaction. Low phone ownership results in patients not receiving appointment reminders or an inability to reschedule missed appointments. Missing clinic follow-up visits are barriers, mentioned by a majority of staff and providers, to completing diagnostic laboratory tests or receiving an HCV diagnosis.

#### Facilitators

Among patients who do not have phones, clinics collaborate with case managers and emergency medical services (EMS) community health paramedics (CHPs) to locate patients to encourage them to keep their appointments and come to the clinic, so they can receive their diagnosis in a timely manner. Providers set the patient up for success by determining patient readiness prior to offering treatment.

“Compliance with the meds is the big issue. So, if we have patients that are just straight up non-compliant with like any meds or we know that, we’re not going to be able to find these people, I just defer it until a different time…But, if I have someone that’s really working at getting the labs with follow up, then I think that that encourages me to really work a little harder to get them to the places they need to be. But a lot of times, just having those labs on file and just [saying], whenever you’re ready, we’ve got what you need lab wise, that’s pretty much it.” (Provider)

Operational facilitators include the use of an HCV group on the clinic’s HIPAA compliant communication platform, on which providers can message other providers who treat HCV and get timely answers to case-based questions or readily available consultations with hepatology specialists. Additionally, having on-site laboratory testing reduces the likelihood of patients leaving the clinic without getting the necessary labs drawn to offer treatment.

### Treatment accepted

#### Barriers

Several barriers to accepting treatment are a function of the nature of this population, such as patients not feeling ready, low health literacy, competing medical priorities, and living with untreated mental health conditions and/or substance use disorders, resulting in patients not returning to the clinic to accept treatment. Accepting treatment can be a common drop off point for patients who are asymptomatic and feel that they do not need treatment, or who have other competing psychosocial priorities, or face additional barriers to remaining engaged in care.

“[Hepatitis C] just needs more exposure. Like, when they first told me I had it, I didn’t really take it seriously, I didn’t think it was that serious, hepatitis C, and so I didn’t really care if I got medical help or not.” (Patient)

#### Facilitators

Many patients mentioned strong social networks influencing them towards undergoing treatment in numerous ways, such as friends who encourage them to get treated, friends informing them that treatment is easy and within reach, a patient wanting to prove to family members they are taking responsibility for their health, watching loved ones with HCV suffer or die.

“I said yeah, I got to look into this and get [my hepatitis C] cured. I’m not ready to die. (Patient who reached SVR12)

Educating patients on the consequences of untreated HCV increases their motivation to get cured. Providers and care coordinators can make the treatment regimen more acceptable to patients by making it more manageable for them, breaking down the care cascade into discrete pieces, and working with patients to create a specific plan for treatment and medication storage.

### Getting funding for treatment

#### Barriers

The majority of patients treated at CHC HCH sites lack health insurance. Patients on the county-based medical access program (MAP) for the uninsured, which includes many PEH and PWID, commonly experience unnoticed lapses in coverage, which interferes with completion of requisite medical appointments or laboratory tests prior to initiating treatment. For patients covered by MAP, their DAA medications must be covered by the prescription access program (PAP), which is through a separate application process from MAP. Completing necessary PAP paperwork can be cumbersome and confusing to some patients who may conflate one assistance program for another. Verifying information for this paperwork, such as getting a letter of no income or proof of residency, can be challenging, given how difficult it is for this population to return to clinic, unreliable communication methods, and lack of necessary identification or income documentation. Additionally, the strict requirements of prescription assistance programs can impede access to funding for treatment. While patients on Medicare or Medicaid are not eligible for PAP, they do have access to DAAs under these programs. However, this access often requires prior authorization or other bureaucratic steps that are confusing and time-consuming for a few providers and clinic staff and can delay access to treatment for patients.

“…the process of how getting their medication that just takes forever. It feels like forever…Then the patient’s also left [wondering], what’s going on, you told me to sign this paper, and I was going to get these medications and, where are they? And the patient gets angry, the patient [is] like what’s going on, it’s been so long.” (Clinic Staff)

#### Facilitators

Clinic staff make the funding paperwork process easier for patients by getting patients’ signatures and information at the time of treatment acceptance. Though MAP does not cover the cost of medication, it covers medical care and labs and serves as a facilitator to medication access by guaranteeing patients’ DAAs are covered by prescription assistance programs. On the operational side, specifying which labs are required by each payor and ensuring this information is known and shared across clinic providers and clinic staff further streamlines the funding application process.

### Treatment initiated

#### Barriers

Once a diagnosis is received, low health literacy can impact a patient initiating treatment and adhering to medication. PEH and PWID often experience competing medical priorities, reducing the likelihood of sensing any urgency to undergo treatment for their HCV or pick up medication. Alternatively, patients who are motivated to initiate treatment face transportation, communication, and systemic obstacles to get to clinic to pick up their medication.

“Okay, and then do you have medication, so you know we’ll get you seen, but then do you have insurance coverage? You know, do you have insurance coverage to get your– to pick up your meds, and they’re like no I lost my ID and everything. So, they don’t have ID or insurance or so it’s like, okay, now we have to get you ID.” (Nurse)

#### Facilitators

Initiating treatment is facilitated by clinic staff collaborating with the pharmacy department and external partners to improve patient access, by either couriering medication to locations patients can readily access, for example, the clinic they prefer to go to, or through direct delivery to patients by members of the Street Team, Mobile Team, or community health workers.

Patient oriented care, here defined by active encouragement and support of patients by clinic staff, was mentioned by many clinic staff and providers to facilitate acceptance and initiation of treatment by building confidence and bolstering engagement in care throughout the care cascade. Co-locating HCV treatment with corollary care, such as medication assisted treatment with buprenorphine for opioid use disorder, or by co-locating clinics where psychosocial resources, which are perceived as more pressing needs such as free meals or applying for housing, are being provided, increases the likelihood of patients engaging with clinics, including picking up their HCV medication and getting labs drawn.

### End of first month

#### Barriers

Once a patient receives funding for treatment, clinic operations may raise barriers to patients’ ability to adhere to their medication regimen or get necessary labs drawn. For example, patients may be confused about where to pick up their medication, especially if they frequent more than one HCH clinic or the pick-up location is not their usual clinic, or they may not understand the importance of communicating with their provider about side effects or perceived side effects. Furthermore, the long wait times of the CHC’s centralized call center makes it hard for patients to connect directly with an individual clinic. As a result, patients may decide to stop taking their medication on their own if they experience side effects and if they cannot or do not connect with their medical team in a timely manner.

“You can’t get a hold of anybody, you’ll be on the phone for hours, usually. Unless you know the number of the person linked to the, which I always end up getting stuff stolen so I don’t always have the numbers. But if the number you call, that one main number, it’s very difficult.” (Patient)

#### Facilitators

At the clinic level, staff and providers take many actions to facilitate patients reaching this point in the care cascade, including offering mid-way check-in appointments, giving patients the opportunity to start treatment when they are ready, and emphasizing the importance of medication adherence. Rapid replacement of lost or stolen medications through PAP programs provides additional external support to medication adherence.

“I do also try to address one of the 2 or 3 contingencies that come up, namely, it’s very common with patients, especially at Care Connections, if you lose your medicines – if you do, please don’t wait until the next appointment to tell me, please come here right away and we’ll talk to the drug company and usually we’ll get you a new bottle. So, I talk about that contingency. I talk about if [their medication] gets lost or stolen.” (Clinic Staff)

Additionally, strengthening the patient and provider relationship while continuously providing support by breaking down care into steps manageable by the patient, may increase patient communication with the clinic.

“One size doesn’t fit all. The traditional way we think medical care should be delivered, which is that doctors do medical things, has now evolved into, like, we need to address the whole person, their mental health needs, their emotional needs, their spiritual needs, while addressing the physical health needs. And deliver it in a way that is compassionate and patient-centered…We really believe that the patient has a lot of challenges in general and we shouldn’t expect them to jump through hoops, and do all these things that many people with phones, and cars, and not a lot of other issues can handle. And so that’s why we do the way we practice hep C.” (Provider)

The consistency of the Mobile Team’s location schedule increases the likelihood of walk-ins to clinics that are walk-in based, since patients will know when and where the clinic will be, especially important for patients without phones. The HCH clinics’ flexibility with scheduling and lateness allows patients to feel comfortable calling or coming into clinic if they experience side effects before stopping medication.

### End of treatment

#### Barriers

Patient level barriers to completing treatment are centered around low health literacy, lapses in medication adherence, and failure to return to the clinic for end of treatment (EOT) labs. Additionally, many participants mentioned the lack of safe storage to prevent medication loss or theft, frequent jail or hospital stays, and late medication refills resulting in adherence lapses or preventing patients from completing treatment.

“They’re experiencing homelessness, they lose their medication, their medication gets stolen.” (Clinic Staff)

#### Facilitators

The same facilitators to medication access and patient engagement in initiating treatment are leveraged by clinics and staff to help patients adhere to their medication and reach treatment completion.

### SVR12

#### Barriers

The need for laboratory tests to assess SVR12 to determine cure is not always understood by patients with low health literacy, so they do not return to clinic to get the blood drawn for laboratory tests. Patients who do understand the importance of SVR12 labs, may not always remember to come back for labs. Additionally, some providers may not sufficiently emphasize the importance of the SVR12 labs, knowing there is a high correlation of earlier undetectable viral loads with cure.

“But for people that have finished treatment, a lot of it has to do with the stability of their social setting. Because it’s hard to remember to come back 3 months after you’ve finished treatment to get labs drawn when you’re still out on the streets.” (Clinic Staff)

#### Facilitators

For some patients, motivation to verify HCV cure drives completion of SVR12 labs. For clinics, getting SVR12 labs is made easier by providers ordering SVR12 labs ahead of time, so they can be completed whenever the patient next comes into the clinic beyond 3 months of completing treatment. Many clinic staff members reported celebrating patient wins such as HCV cure boosting morale of both patients and staff.

### Incorporating qualitative findings into site-specific protocol design workshops

These qualitative findings were incorporated into the human-centered design workshops to design site-specific, simplified treatment protocols. Even though, in the first workshop, numerous barriers to getting patients into clinic and linked to care were identified, during the human-centered design workshops, clinic teams focused on operational clinic-level barriers they had the ability to modify along the HCV care cascade, which starts at diagnosis, after they have been screened and linked to care. The HCV care cascade starts at diagnosis because that’s when the treatment process within the clinic starts. As a result of using the human-centered design approach, clinic staff, who participated in the design workshops, garnered how an extensive volume of factors related to getting the patient into the clinic has repercussions down the line of the HCV care cascade that can be impacted operationally. Not all barriers, such as a patient going to jail or being hospitalized while on treatment, and losing access to their medication as a result, could be modified by clinic staff. Existing facilitators informed actions to leverage and emphasize, while modifiable barriers invited opportunities to propose solutions to be incorporated into the site-specific treatment protocols.

**Fig. 4:**
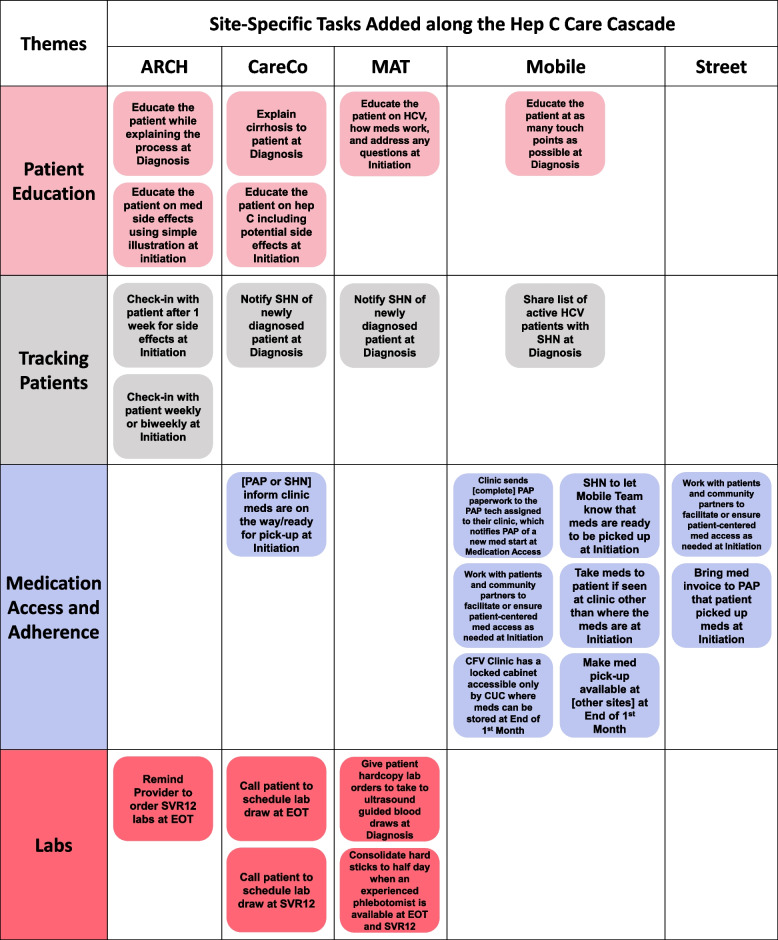
Themes of site-specific tasks added along the Hep C care cascade by clinic

The protocol variations across sites were operational differences in how teams accomplish tasks and who takes ownership of the tasks along the care cascade, taking into consideration varying staffing ratios, physical spaces, team dynamics, and characteristics of the patients that frequent each site. Actionable solutions incorporated into each site-specific protocol pertained to patient education, tracking patients along the care cascade, improving medication access and adherence, and ensuring patients return for necessary labs (Fig. 4). The Mobile Team designed a singular protocol for all the three mobile sites (CFV, Esperanza, and Sunrise).

Clinic teams incorporated medication adherence counseling by ensuring patient HCV education was woven into the workflows. Following patients along the HCV care cascade also aids in ensuring patients’ medication access and adherence, which was especially emphasized on the Mobile and Street Team workflows. Clinics incorporated a variety of methods to start tracking patients after treatment has been accepted such as augmented existing staff roles to leverage existing facilitators and optimize patient access to medications. All the clinic teams, except the ARCH clinic which has a care coordinator, chose to leverage the pre-existing resource of the CHC’s Sexual Health Team’s patient navigators (SHNs) to support patients through prescription access programs to obtain medications for uninsured patients. Care coordination and education helps patients remain engaged in care with the single goal of being cured.

## Discussion

This qualitative study identified barriers and facilitators to HCV treatment at primary care clinics within our local community health center’s Health Care for the Homeless program to inform the design of simplified, site-specific HCV treatment protocols for serving vulnerable populations in Austin, Texas. Barriers and facilitators to each step along the care cascade were identified, in order to reduce their negative impact or leverage their positive contribution to care. Participants were interested and had extensive knowledge and ideas to contribute as study goals melded with clinic objectives to improve care for people with HCV, especially among PEH and PWID.

Barriers encountered at each step along the care cascade are a result of the vulnerabilities uniquely experienced by this population, such as competing priorities, untreated mental health conditions and/or substance use disorders, living environment, unreliable communication, and inability of mental capacity to sufficiently plan to seek care in the face of unreliable transportation, where patients experience stigmatizing behavior, as also demonstrated in other studies [9, 10, 12, 23]. All these characteristics of the population conspire to result in high “no show” rates among this population that can compromise patient care when patients miss their appointments and do not receive an HCV diagnosis, pick up medications, or complete laboratory tests [1, 9, 12].

Though studies have shown that 40% of people are unaware of their HCV status, our results demonstrate that patients often do know their HCV status but face a host of barriers that can result in patients not seeking care despite knowing their status including not knowing that treatment is available and accessible to them [9, 12, 23–25]. Actionable solutions to further bolster engagement in treatment clustered around enhancing and streamlining patient education, facilitating medication access, and care coordination. Actionable solutions added to the care cascade spanned across all PRISM domains, from system-level collaboration with other departments within our community health center (e.g., sexual health navigators) to working with external community partners (e.g., emergency medical services) who are key stakeholders to facilitate medication access [12, 25]. Clinics without a dedicated care coordinator augmented existing clinic roles and incorporated utilizing sexual health navigators into their protocols to help track and support patients throughout the care cascade. For some patients, high motivation for HCV treatment is a significant contributor to overcoming these barriers.

Despite these barriers, social resources co-located with the clinics and corollary care (e.g., buprenorphine for opioid use disorder), compounded by the supportive clinical environment, motivate patients to return to clinic at all steps along the care cascade [25–27]. Existing operational facilitators to creating a supportive care environment include being patient-oriented, teamwork, strong communication, and flexibility among and across clinics. Clinics do not turn away patients who are late, accept patients who walk-in and utilize a standby list, set up clinics at various locations around town, and collaborate with external organizations, such as the emergency medical services community health paramedics, to not only be on the lookout for patients but also meet the patients where they are [12, 25, 26].

Through interviews and human-centered workshops, we obtained a multi-level perspective across an expansive breadth of clinics not yet discussed together in literature. Anchoring the barriers and facilitators along the HCV care cascade allowed the clinic teams to identify actionable solutions to break down barriers. The end of the first month was noted as a critical point in the HCV care cascade where both barriers and facilitators were most quantifiably weighted. Several barriers that were frequently identified in interviews and workshops have been obviated by the simplified treatment algorithm itself, which removed some steps that had earlier been required and were often difficult for this population in particular to accomplish, such as requiring appointments and labs at the end of the first month of treatment [8].

Stressing the importance of coming back to have sustained virologic response 12 weeks post-treatment labs drawn to determine cure was emphasized in the workshops as an important step in the care cascade that could be facilitated by the support of clinic staff and availability of on-site laboratory testing [25–27]. There was an identified need to increase HCV patient knowledge on the importance of sustained virologic response 12 weeks post-treatment labs or calling the clinic when experiencing side effects before stopping medication to increase patients reaching cure [9, 27, 28]. Increasing patient knowledge includes continuing to make each step as easy as possible for the patients by leveraging how providers break down medical care into bite size pieces for the patients to better understand treatment and the vital steps of the process since many patients are motivated by seeing they have been cured [9].

### Value of a human-centered design approach

By taking a ground-up systematic approach, rather than a top-down approach across all sites, each clinic team and staff were empowered to provide input into designing their own site-specific protocols. Inviting staff across all roles to participate in their site-specific design workshops to co-design the simplified, site-specific protocols garnered buy-in and ownership of the protocols at each site. Buy-in was also garnered by the study team’s interest in observing and understanding clinic processes, and building relationships with clinic teams by their presence in clinic, in preparation for the second phase of the study.

## Limitations

Our study has its limitations. There was the risk of clinic staff being hesitant to fully disclose and discuss operational barriers for fear that supervisors or teammates would prefer not to disclose some of the barriers. To encourage staff to be forthcoming, interviews were conducted in private spaces, out of ear shot of others, and staff were assured that information would be anonymized. Study staff spent time in clinic, to observe and learn clinic practices, build relationships, and communicate the intent of the study so clinic staff can be more comfortable engaging in more open and honest dialogue. Additionally, due to the COVID-19 pandemic, interviews required flexibility and participants were offered in-person or virtual interviews. As the gold standard, in-person interviews allow for a wider read of body language, which may be more limited in virtual interviews conducted over Zoom. On the other hand, offering remote interviews may have led to a higher response rate among those who preferred a remote interview. Despite some staff in non-provider roles attesting during interviews or design workshops to having limited or no knowledge of HCV and the HCV treatment process, they were usually able to draw on their experience with general treatment processes and caring for this particular patient population to offer information and perspectives that were applicable to the HCV treatment process as well, garnering buy-in from all clinic staff for the delivery phase of the human-centered design approach.

## Conclusions

Though HCV treatment by primary care providers has been proven effective, hesitancy to treat PEH and PWID remains a barrier to treating this population and eliminating HCV worldwide [9, 10]. The Erase Hep C study aims to minimize this barrier and make HCV treatment more accessible locally to high-risk, vulnerable patient populations, and easier to incorporate into busy, primary care provider workflows, including in non-traditional clinic settings.

The finding that co-morbidities and other medical and psychosocial priorities, transportation, and communication issues can be barriers to HCV treatment may be generalizable to the treatment of other diseases in this population of PEH and PWID. Additionally, findings may be generalized to PEH and PWID with HCV in other settings. System-level findings, such as confusing processes to access medication and long wait times to acquire medication may be relevant to other community health centers serving vulnerable populations in other settings in the US. On the other hand, these may not be relevant, emphasizing the importance of locally contextualized treatment protocols.

We describe a method to incorporate staff and patient knowledge and experience to design contextualized HCV protocols for treating vulnerable populations in non-traditional clinic settings. Through a collaborative approach, these protocols will be integrated into clinic workflows and providers will be trained on simplified HCV treatment. In the second phase of the Erase Hep C study, the site-specific protocols developed in the design workshops will be implemented and patients will be enrolled into our single-arm trial with the aim of at least 75% of our study participants achieving sustained virologic response 12 weeks post-treatment. In this next phase of our study, we will conduct the last two steps of the human-centered design approach by delivering and implementing the site-specific treatment protocols, and measuring both clinical and implementation outcomes [14]. Ultimately, this approach could serve as a model for future implementation research aiming to develop and implement contextualized treatment models for other conditions in vulnerable populations.

### Supplementary Information

**Additional file 1.** Standards for Reporting Qualitative Research (SRQR)* Checklist for Identifying barriers and facilitators along the Hepatitis C care cascade to inform human-centered design of contextualized treatment protocols for vulnerable populations in Austin, Texas: a qualitative study.

**Additional file 2.** Erase Hep C Phase 1 – Patient Interview Guide.

**Additional file 3.** Erase Hep C Phase 1 – Clinic Staff Interview Guide.

**Additional file 4.** Erase Hep C Phase 1 – External Testing Organizations Interview Guide.

## Data Availability

Qualitative interview guides can be made available upon request.

## Abbreviations

CareCo: Care Connections Clinic
CFV: Community First! Village
CHC: Community health center
CHP: Community health paramedic
CHW: Community health worker
DAA: Direct acting antivirals
EMS: Emergency medical services
EOT: End of treatment
HCH: Health Care for the Homeless
HCV: Hepatitis C virus
LCSW: Licensed clinical social worker
MA: Medical assistant
MAC: Medical administrative clerk
MAP: Medical access program
MAT: Medication-assisted therapy
PAP: Patient assistance program
PEH: People experiencing homelessness
PRISM: Practical, Robust Implementation and Sustainability Model
PWID: People who inject drugs
RN: Registered nurse
SHN: Sexual health navigator
SRQR: Standards for Reporting Qualitative Research
SVR12: Sustained virologic response 12 weeks post-treatment
US: United States
