# Supplementary material for: Identifying barriers and facilitators along the hepatitis C care cascade to inform human-centered design of contextualized treatment protocols for vulnerable populations in Austin, Texas: a qualitative study
Source: Implement Sci Commun. 2023 Aug 17;4:98. doi: 10.1186/s43058-023-00484-6 (PMC10436407; doi:10.1186/s43058-023-00484-6)
Supplement: Supplementary file 2 — Additional file 2. Erase Hep C Phase 1 – Patient Interview Guide. [file 43058_2023_484_MOESM2_ESM.docx]

Introduction: Hello, my name is ____, and I am working with UT Austin Dell Medical School and CommUnityCare on a study about treating hep C and we’d like to get input from patients. May I ask you some questions to learn about your experience at this clinic and with hep C?

Your privacy is protected. We will keep anything you say confidential, stored in a secure location, and not share it with anyone outside our research study team.

Your participation is voluntary. If you choose not to participate, this will not affect the health care you get with CommUnityCare. If you do agree to be interviewed, I would like to record this interview to ensure we accurately capture your thoughts. This interview will take about 30-45 minutes and you will receive $10 for participating in this study. Is it okay to continue? Do I have your permission to record this interview? [Request patient to sign 2 consent forms.]

Clinic Name: ___

Let me ask you about the clinic in general.

1. **Why do you come to this clinic?** [OC], [PC], [EE]
   1. What do you like about coming to this clinic? Is there anything hard about getting care at this clinic? What was easy? [OC], [PC], [EE]
   2. How does it compare to other clinics or healthcare spaces you’ve been to?

- Probes:
  - Knowledge and Perceptions – time constraints, financial costs, staff interactions
  - Internal Processes – scheduling, getting an appointment, talking to a live person on the phone, navigating the system, getting insurance
  - Clinic Environment– space, location, clinic hours, wait times
  - External – transportation, weather
  - Clinic – space, location, clinic hours, appointments, wait times
  - Staff – relationship with providers and clinic staff
  - Additional resources

1. **Tell me about when you have to come back to the clinic. You come to the clinic, see [Dr ___], you have to come back, how does that go?** [OC], [PC], [EE]

- Probes:
  - How do you remember when and where to come back for appointments?
    - Are you given an appointment reminder card? Do you get a phone call? Does someone help you?

1. **Can you tell me what you know about hep C?** [PC], [SP]

- Probes:
  - - How can it impact someone, how can you get it, can it be cured, etc.
  - How did you find out about that?

1. **Tell me about your experience with getting tested for hep C.** [OC], [PC], [EE]

- Probes:
  - Was it hard or easy?
  - How did you know about where to get tested?
  - Where did you get tested?
  - How long ago was it?
  - Why did you get tested?
  - Did anyone counsel you on risk of transmission and passing it on to someone else?
  - What did you think when you got your result? How did you feel? What did you do?

1. **Talk to me about hep C treatment. Have you been on treatment?** [PC], [EE]

Not been on treatment

1. **Tell me about why you haven’t been on treatment?** [PC], [EE]

- Probes:
  - - Has anybody offered you treatment?
    - Do you know why you weren’t offered treatment?
    - What would have to change for you to consider treatment?

On or been on treatment

1. **Tell me about the treatment process.** [OC], [PC], [EE]
   - 1. What did your doctor tell you about treatment?
        - What was helpful?
        - What did you have questions or concerns about?
     2. Why did you decide to get treatment?
     3. What was it like being on treatment?
        - What was the best part?
        - What was the biggest challenge?
2. **Have you been on treatment for other conditions? How did that compare to being on hep C treatment?**
3. **What would you recommend to us to make treatment easier for others?** [OC], [EE]

Everyone

1. **Is there anything you’d like to add that you haven’t told me about?**

We’d like to make sure we speak with people of different backgrounds, could you tell me:

What is your age?

What gender do you identify as?

Male

Female

Trans Male

Trans Female

Another Gender or Non-Binary

Don’t Know

Not Disclosing

What is your race? (check all that apply)

White

Black/African American

Asian

Native American/Alaska Native

Native Hawaiian

Other Pacific Islander

Not Disclosing

Do you identify as Hispanic or Non-Hispanic?

Hispanic

Non-Hispanic

What is your housing status?

Doubling up

Homeless shelter

Other

Permanent supportive housing

Street

Transitional

Unknown

Have you ever injected drugs?

Yes

No

Thank you for taking the time to speak with me.

| **PRISM Codes** | **PRISM Domains** |
| --- | --- |
| [OC] | Organization Characteristics |
| [OC/S] | Organization Characteristics/Staff |
| [PC] | Patient Characteristics |
| [ISI] | Implementation and Sustainability Infrastructure |
| [EE] | External Environment |
| [SP] | Side Project (Barriers to Completing Treatment) |
