# Supplementary material for: Identifying barriers and facilitators along the hepatitis C care cascade to inform human-centered design of contextualized treatment protocols for vulnerable populations in Austin, Texas: a qualitative study
Source: Implement Sci Commun. 2023 Aug 17;4:98. doi: 10.1186/s43058-023-00484-6 (PMC10436407; doi:10.1186/s43058-023-00484-6)
Supplement: Supplementary file 3 — Additional file 3. Erase Hep C Phase 1 – Clinic Staff Interview Guide. [file 43058_2023_484_MOESM3_ESM.docx]

Introduction: Hello, my name is ____, and I am working with UT Austin Dell Medical School and CommUnityCare on a study about treating hep C in high-risk, vulnerable populations, such as persons experiencing homelessness and persons who inject drugs. May I ask you some questions to identify the facilitators and barriers in treating hep C in the CUC patient population?

Your privacy is protected. We will keep anything you say confidential, stored in a secure location, and not share it with anyone outside our research study team.

Your participation is voluntary. If you choose not to participate, this will not affect your relationship with CommUnityCare. If you do agree to be interviewed, I would like to record this interview to ensure we accurately capture your thoughts. This interview will take about 30-45 minutes. Do I have your permission to record this interview? [Request staff to sign two consent forms.]

Clinic Name: ___

*Ask roles indicated in green questions in green and blue. Ask roles indicated in blue ONLY questions in blue.*

For providers and non-providers:

1. **What is your role at this clinic?** [OC/S], [ISI]
2. **What does a typical day look like for you?**

For non-providers (RN, MA, MAC, CHW, LCSW, Behavior Health Consultant, Case Manager, Referral Team) AND (Central Pharmacist, Nurse Manager, Practice Administrator, C-Level Leadership, PAP Coordinator, HCH Eligbility Worker):

1. **Does your role involve anything to do with caring for patients with hep C? (For C-Level Leadership: Given your leadership role, tell me how CUC treats hepatitis C?)** [OC/S]

IF YES: Tell me about your experience caring for a patient with hep C.

- 1. How do you support the care of a patient with hep C? [OC/S]
  2. Can you describe your workflow? [OC], [OC/S]
  3. What’s easy?
  4. What’s challenging or takes more time than you think it should?
  5. How would you make the hep C workflow process simpler and more efficient? [OC], [OC/S], [ISI]

IF NO: Let’s talk about your role and CUC workflows in general.

- 1. What’s easy to do in your role?
  2. What’s challenging or takes more time than you think it should?
  3. How would you make the general clinic workflow simpler and more efficient? [OC], [OC/S], [ISI]

For providers and non-providers:

Let’s talk about hep C.

1. **Describe the screening process for hep C.**

- Probes
  - What part of this process goes as planned?
  - What parts of this process are most challenging?

1. **Describe the testing process for hep C.**

- Probes
  - What part of this process goes as planned?
  - What parts of this process are most challenging?

1. **Do you have patients coming to you who have already been diagnosed with hep C? If so, how do you find out about it and how do you link them to care?** [PC], [EE]
2. **Now, let’s talk about treatment initiation.**
   1. Do you ever not offer a patient hep C treatment? Why not? [PC], [EE]

- Probe: current substance use, life-limiting condition, non-compliance with appointments, medication
  1. How do you start a patient on treatment? [OC], [OC/S]
     1. Who else supports the process? [OC/S]

1. **Tell me about a time it was particularly challenging to get a patient started on treatment. (For C-Level Leadership: Thinking about from a system level)** [OC], [OC/S], [ISI], [EE]
   1. How common are these barriers for other hep C patients? What other barriers do you see? [OC], [OC/S], [EE]
   2. What have you seen work well to overcome these barriers? [OC], [OC/S], [ISI], [EE]
   3. What do you think works well? [OC], [OC/S], [ISI]

- Probe: genotyping, ultrasound for fibroscan, documentation, etc.
- Note what they are doing formally and informally

1. **What motivates patients to start treatment?** [OC/S], [PC], [EE], [SP]

- Probe:
  - Do you know this because the patient tells you?

1. **What delays patients from starting treatment? I mean from the time a patient is told they have hep C to the time they pick up of their first treatment dose?** [OC], [PC], [EE], [SP]

- Probe:
  - Do you know this because the patient tells you?

Let’s talk about staying on and completing treatment.

1. **Tell me about a patient who was able to stay on and complete treatment. What helped them stay on and complete their hep C treatment?** [OC/S], [PC], [EE], [SP]
   1. What do you do to support your patients to stay on and complete their hep C treatment?
   2. Does the clinic do anything to help patients stay on their treatment? [OC]

- Probe: clinic internal organization
  1. Do all patients complete treatment? [PC]

1. **Tell me about a time it was particularly challenging to get a patient to stay on and complete treatment.** [OC], [PC], [EE], [SP]
   1. What can help patients to complete treatment? [OC], [ISI], [SP]
2. **What motivates a patient to come back for an SVR12 lab?** [OC], [ISI], [SP]
   1. What makes it hard for a patient to come back for an SVR12 lab?
   2. How can we encourage patients to come back for SVR12 labs?
3. **How do you depend on your team members?**
4. **How does documentation factor into the workflow of the hep C treatment process?** [OC], [ISI]
5. **Are patients getting reinfected after completing treatment?** [PC]
   1. Why are patients getting reinfected?
   2. What can CUC do to help prevent reinfection? [OC], [ISI]
6. **I don’t have anymore questions, is there anything you would like to add?**

For the **Central Pharmacist** – skip screening and testing questions

1. What are the CUC processes for patients to get their hep C medication?
2. Can you describe the documentation process?
3. From your perspective what do you see as barriers to patients getting their hep C medication?
4. What could be done to make it easier?

For the **C-Level Leadership**

1. Can you describe an example of a new program that was rolled out at CUC that was successfully implemented?

- Probe: What are the steps required when implementing a new program?

1. In your role, what do you have to do to ensure successful implementation of a new program?
2. Can you describe when a change in CUC procedures was planned but fell short of its mark?

We’re recording the age, gender, race, and ethnicity of people we interview.

What is your age?

What gender do you identify as?

Male

Female

Trans Male

Trans Female

Another Gender or Non-Binary

Don’t Know

Not Disclosing

What is your race? (check all that apply)

White

Black/African American

Asian

Native American/Alaska Native

Native Hawaiian

Other Pacific Islander

Not Disclosing

Do you identify as Hispanic or Non-Hispanic?

Hispanic

Non-Hispanic

Thank you for your time.

For providers (MD, NP): We would like to interview patients, do you have any suggestions on the best way to identify and coordinate interviews with hep C patients?

| **PRISM Codes** | **PRISM Domains** |
| --- | --- |
| [OC] | Organization Characteristics |
| [OC/S] | Organization Characteristics/Staff |
| [PC] | Patient Characteristics |
| [ISI] | Implementation and Sustainability Infrastructure |
| [EE] | External Environment |
| [SP] | Side Project (Barriers to Completing Treatment) |
