# Supplementary material for: Identifying barriers and facilitators along the hepatitis C care cascade to inform human-centered design of contextualized treatment protocols for vulnerable populations in Austin, Texas: a qualitative study
Source: Implement Sci Commun. 2023 Aug 17;4:98. doi: 10.1186/s43058-023-00484-6 (PMC10436407; doi:10.1186/s43058-023-00484-6)
Supplement: Supplementary file 4 — Additional file 4. Erase Hep C Phase 1 – External Testing Organizations Interview Guide. [file 43058_2023_484_MOESM4_ESM.docx]

Introduction: Hello, my name is ____, and I am working with UT Austin Dell Medical School and CommUnityCare on a study about treating hep C in high-risk, vulnerable populations, such as persons experiencing homelessness and persons who inject drugs. May I ask you some questions to identify the facilitators and barriers in testing and linkage to hep C treatment in Austin?

Your privacy is protected. We will keep anything you say confidential, stored in a secure location, and not share it with anyone outside our research study team.

Your participation is voluntary. If you choose not to participate, this will not affect your relationship with UT Austin Dell Medical School or CommUnityCare. If you do agree to be interviewed, I would like to record this interview to ensure we accurately capture your thoughts. This interview will take about 30-45 minutes. Do I have your permission to record this interview? [Request to sign two consent forms.]

External Testing Organization Name: ___

*Ask external testing organization that treat HCV questions in blue ONLY.*

**I’d like to talk to you about hep C and how [clinic name] is involved in hep C care.**

1. **You’re doing the testing, someone’s positive, how do you communicate this to the patient?**
2. **Then what happens? Do you offer treatment? Do you refer them elsewhere?**
   - Probes:
     - Tell me about the process.
     - Who is involved? What is their role?
     - What works well about the process?
     - What doesn’t work well?
3. **What challenges have you faced in linking patients to care?**
4. **What challenges do you have when you refer someone to CUC?**
5. **How does your experience here compare to your experience at CUC?**
6. **We’re working on making hep C treatment easier for patients to access at CUC by training providers and simplifying treatment protocols. What do you think about that?**
   1. From your perspective, what do you suggest we do to make it easier for your patients to get care with us?
   2. What do you think we can do to make access easier? Make the referral and linkage process easier?

We’re recording the age, gender, race, and ethnicity of people we interview.

What is your age?

What gender do you identify as?

Male

Female

Trans Male

Trans Female

Another Gender or Non-Binary

Don’t Know

Not Disclosing

What is your race? (check all that apply)

White

Black/African American

Asian

Native American/Alaska Native

Native Hawaiian

Other Pacific Islander

Not Disclosing

Do you identify as Hispanic or Non-Hispanic?

Hispanic

Non-Hispanic

Thank you for your time.
